# Evaluating the Interactive Web-Based Program, Activate Your Heart, for Cardiac Rehabilitation Patients: A Pilot Study

**DOI:** 10.2196/jmir.3027

**Published:** 2014-10-29

**Authors:** Christopher Brough, Sally Boyce, Linzy Houchen-Wolloff, Louise Sewell, Sally Singh

**Affiliations:** ^1^Centre for Exercise and Rehabilitation ScienceUniversity Hospitals of Leicester NHS TrustLeicesterUnited Kingdom

**Keywords:** Activate Your Heart, coronary heart disease, cardiac rehabilitation, Internet, Web-based

## Abstract

**Background:**

Conventional cardiac rehabilitation (CR) programs are traditionally based on time-constrained, structured, group-based programs, usually set in hospitals or leisure centers. Uptake for CR remains poor, despite the ongoing evidence demonstrating its benefits. Additional alternative forms of CR are needed. An Internet-based approach may offer an alternative mode of delivering CR that may improve overall uptake. Activate Your Heart (AYH) is a Web-based CR program that has been designed to support individuals with coronary heart disease (CHD).

**Objective:**

The aim of this pilot study was to observe the outcome for participants following the AYH program.

**Methods:**

We conducted a prospective observational trial, recruiting low-risk patients with CHD. Measures of exercise, exercise capacity, using the Incremental Shuttle Walk Test (ISWT), dietary habits, and psychosocial well-being were conducted by a CR specialist at baseline and at 8 weeks following the Web-based intervention.

**Results:**

We recruited 41 participants; 33 completed the program. We documented significant improvements in the ISWT distance (mean change 49.69 meters, SD 68.8, *P*<.001), and Quality of Life (QOL) (mean change 0.28, SD 0.4, *P*<.001). Dietary habits improved with an increased proportion of patients consuming at least 5 portions of fruit and vegetables per day, (22 [71%] to 29 [94%] *P*=.01) and an increased proportion of patients consuming at least 2 portions of oily fish per week (14 [45%] to 21 [68%], *P*=.01). We did not detect changes in anxiety and depression scores or exercise behavior.

**Conclusions:**

We observed important improvements in exercise capacity, QOL, and dietary habits in a group of participants following a Web-based CR program. The program may offer an alternative approach to CR. A mobile version has been developed and we need to conduct further trials to establish its value compared to supervised CR.

## Introduction

Coronary heart disease (CHD) is the world’s leading cause of mortality [1]. Cardiac rehabilitation (CR) has been shown to reduce cardiac mortality by 26% and the incidence of further cardiac events [2]. The World Health Organization defines CR as “activities that favorably influence the underlying cause of the disease and provision of the best possible, physical, mental, and social conditions, so that patients may, by their own efforts, resume as normal a place as possible in the community” [3]. It is characterized by a package of exercise and multidisciplinary education and secondary prevention advice. However, uptake for CR remains poor in many countries [4], despite national targets for uptake. Conventional CR programs are based on time constrained, structured, group-based programs, usually set in hospitals or local leisure centers. A number of barriers for attending CR have been identified, such as distance to travel, lack of transport [5], patients’ reluctance to participate in a group environment, and time constraints due to work commitments [6,7]. Service factors such as lack of capacity, a failure to offer genuine choice of rehabilitation options [8], and poor adoption of technology within CR [9] have also been highlighted as factors contributing to poor uptake.

The limited uptake of conventional CR would suggest that alternative forms of delivering CR need to be developed [10]. Other forms of CR, such as home-based CR, have been tested against conventional CR programs, with the same success [11-13]. These programs offer many of the elements of conventional CR; they are able to provide a program of prescribed exercise and interdisciplinary education, usually as a manual. However, only a very small percentage of those eligible for CR are offered these programs.

There is considerable interest in digital health as a means of delivering care for individuals with long-term conditions of which CHD is an important one, delivering a standard intervention that is not geographically or time restrained. An increasing proportion of retired people are using the Internet [14], reflecting the typical rehabilitation population. In the United Kingdom, 71.9% and 63.7% of males and females aged between 65-74 years respectively have used the Internet [15]. Interestingly, evidence suggests that the number of people seeking health care guidance via the Internet is increasing [16,17].

The use of the Internet allows for greater flexibility as patients are able to complete their CR program at a place and time that suits them. It is also capable of reaching a wider audience, especially those patients that live in rural areas [18]. This growth in Internet use makes it worthy of consideration for intervention development. Previous research has identified the benefits of using Web-based interventions for areas such as weight loss, smoking, physical activity, and reducing depression and anxiety in long-term conditions such as diabetes and asthma [19-23]. Studies have highlighted how Web-based interventions can also help to improve knowledge for patients with chronic health conditions [24].

There is little literature describing initiatives that are relevant to patients with CHD. Studies carried out across Europe, the United States, and Canada have investigated the efficacy of Web-based interventions for those with heart disease. The largest was reported recently in the British Medical Journal [25]; however, it was not a comprehensive rehabilitation program and recruited participants with a broad range of cardio-vascular diseases and was largely inconclusive. An interesting paper [26] reported on a study from Canada that recruited exclusively people post primary percutaneous coronary revascularization (PCI or angioplasty) who were offered a physical activity intervention that was Web-based. The paper reported a benefit in the intervention arm not observed in the control arm, suggesting the potential value of Web-based interventions in this population. An earlier study [27] randomized patients to an Internet-based case management trial providing risk factor management. The intervention group had an increase in weight loss compared to the control group, but no additional benefits were observed in depression scores, minutes of exercise, and dietary habits, the outcomes traditionally associated with cardiac rehabilitation. Another small pilot study (n=15) in Canada compared an online comprehensive CR program with standard CR [28], which showed positive benefits for patients by increasing exercise levels and modifying lifestyle behaviors.

There may also be benefits to the service, releasing capacity for CR specialists to manage more complex patients in conventional CR, as well as providing additional choice for those unwilling to do standard CR [27].

Over the last few years, we have developed and refined an online comprehensive CR program, Activate Your Heart (AYH). The aim of this study was to observe the impact on exercise performance and health-related quality of life on patients who completed AYH.

## Method

### Overview

This was a prospective observational study. Patients were recruited from those referred to CR at the University Hospitals of Leicester NHS Trust. Assessments by a CR specialist were conducted at baseline and after 8 weeks of following AYH. We recruited patients with CHD having one or more of the following: percutaneous coronary intervention (PCI) in the last 3 months, undergone coronary artery bypass graft (CABG) in the last 3 months, or were medically managed for their CHD. To identify low-risk patients, a prospective threshold of at least level 7 (420 meters) of the Incremental Shuttle Walking Test (ISWT) [29] was agreed. Exclusion criteria were unstable angina, significant anxiety/depression ≥11 on Hospital Anxiety Depression Scale (HADS) [30], moderate-severe left ventricular dysfunction, inability to perform physical activity due to significant comorbidities, such as severe arthritis, neurological disorders, psychiatric disorders, and not being computer literate.

### Intervention

**Figure 1:**
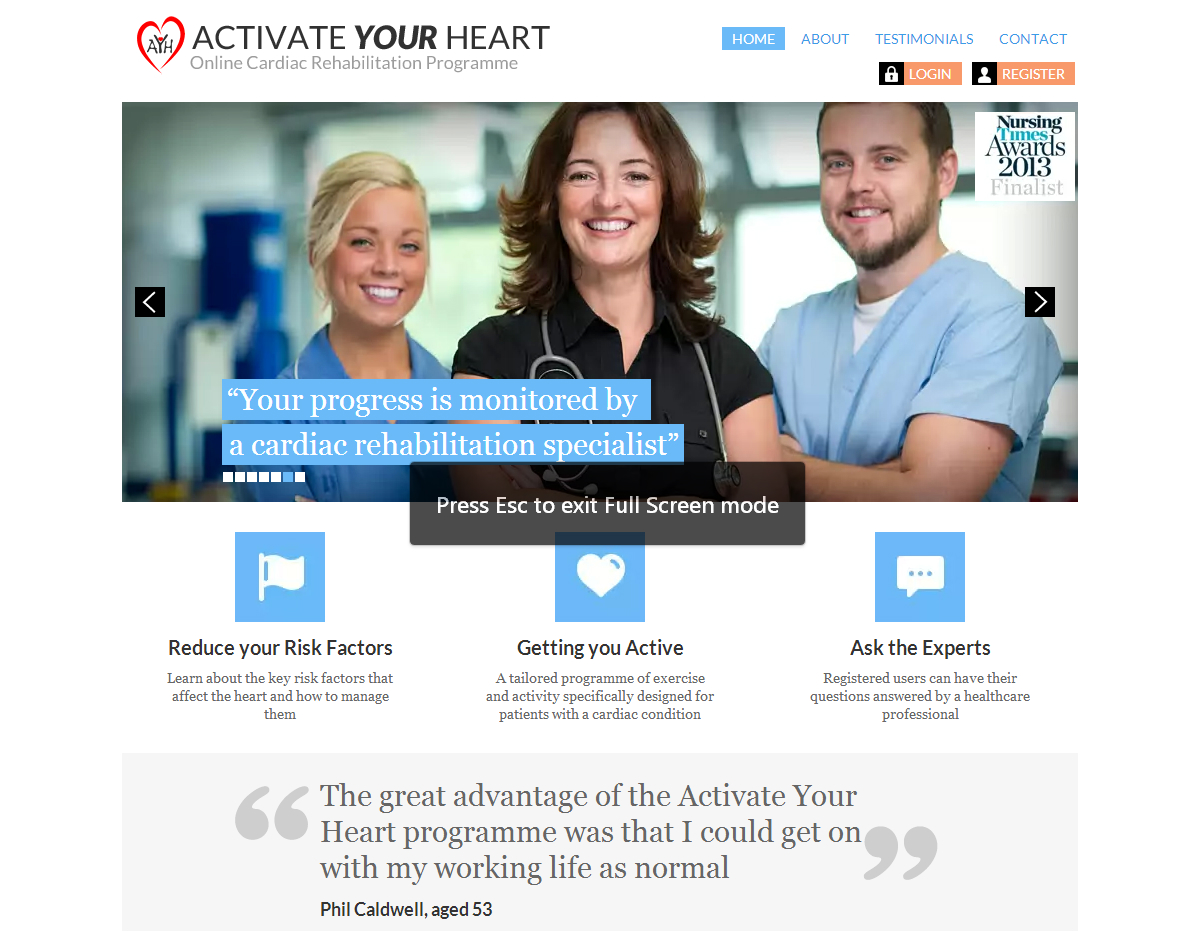
Activate Your Heart homepage.

**Figure 2:**
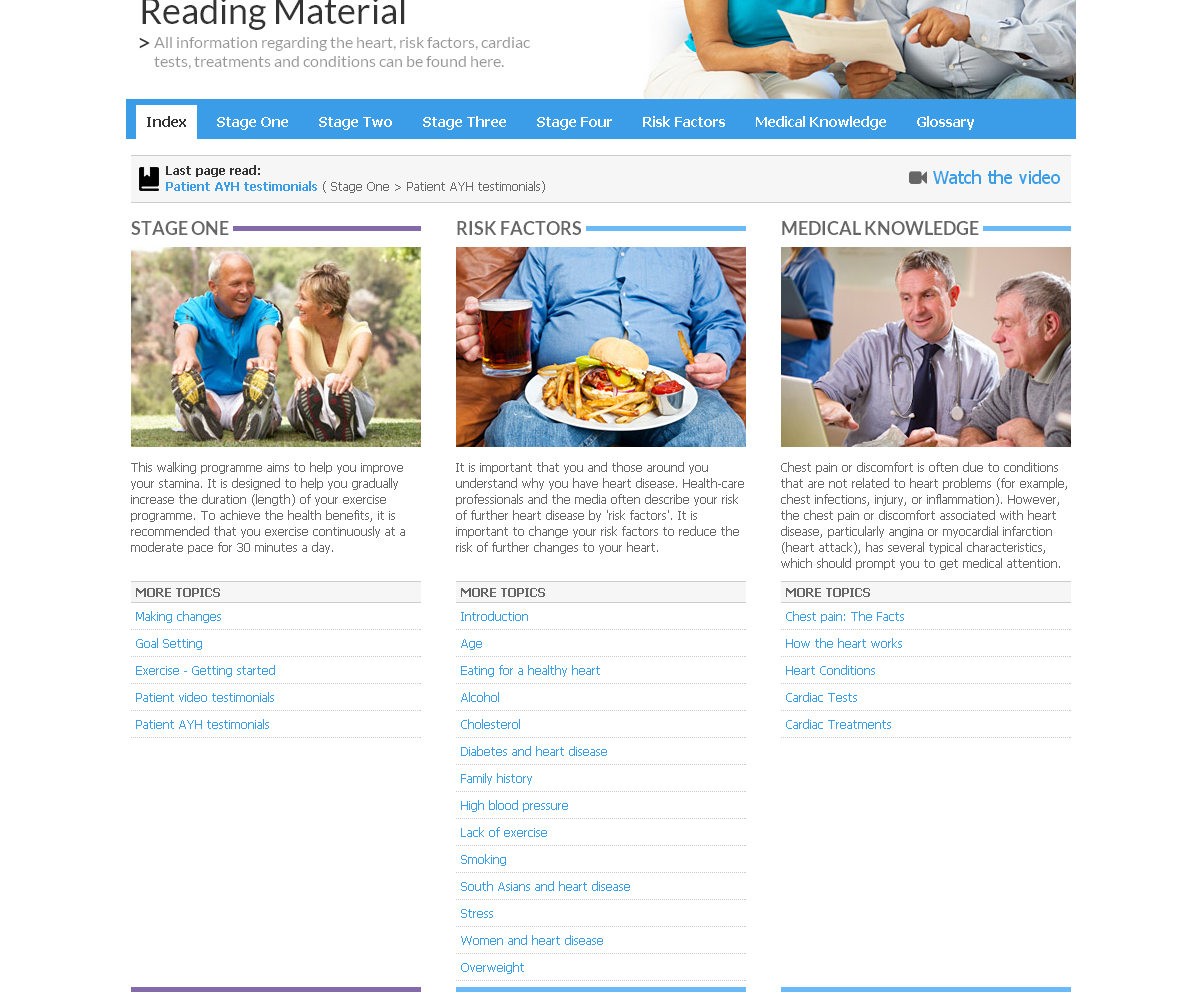
Reading material section.

AYH (Figure 1) is an interactive Web-based CR program that offers comprehensive secondary prevention education together with access to CR specialists via a private messaging facility. It was developed by CR specialists and patients who shaped and informed the final product, at the University Hospitals of Leicester NHS Trust. The program is password protected; each participant was given their own unique password to access the AYH program. All participants were able to record and monitor their exercises, and participate with interactive, secondary prevention advice to promote healthy lifestyle changes and reduce risk factors for CHD, via the website (Figure 2).

**Figure 3:**
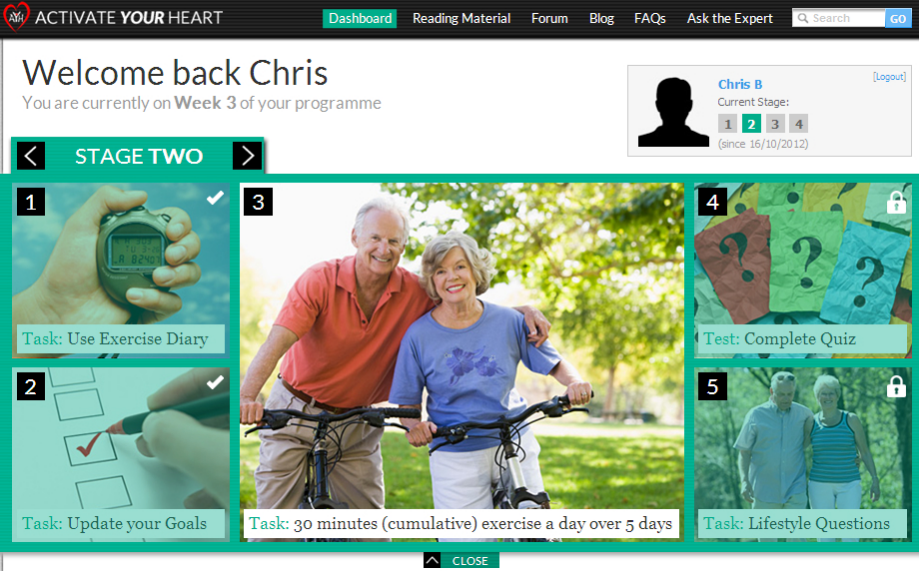
Part of the dashboard showing what tasks need to be completed in stage 2.

The AYH program had been structured so as to guide the user through four stages that each have specific tasks the user needs to achieve before progressing to the next stage (Figure 3). Tasks included creating and updating their own short-term goals, completing knowledge tests on CHD and the risk factors, and reading specific topics such as goal setting and making lifestyle changes. The educational reading material includes videos and covers topics such as anatomy and physiology of the heart, CHD, risk factors for CHD, cardiac tests, and treatments for CHD (Figure 2).

In Stage 1, participants were asked to do a multiple choice questionnaire to establish their knowledge regarding the principles of exercising safely. A score of 80% was set as a threshold to ensure understanding of these principles. In Stages 2-4, participants were required to record all their exercises in an exercise diary. During Stage 2, participants were advised to record a cumulative total of 30 minutes of exercise 5 days out of 7. The intensity of the exercise was based on their performance on the baseline exercise tests and prescribed at 60-80% of baseline performance. In Stage 3, this was amended to 30 minutes of continuous exercise. Finally, in Stage 4, participants were required to record at least 30 minutes of at least moderate exercise 5 days out of 7, in order to fulfill the national requirements for exercise. There is also interactivity around diet and weight management, stress management, and smoking cessation, if appropriate. For smokers, a cost calculator was developed that would calculate how much the user had spent or saved since starting the program. This was delivered along with advice and support to stop smoking.

Other features embedded within the AYH program included a forum where patients were also able to share views and experiences with other program users, a blog, and a frequently asked questions section. The forum was monitored and moderated, as necessary, by CR specialists. In addition patients were able to communicate privately with a CR specialist via the Ask the Expert messaging facility. All queries were answered by a CR specialist within 48 hours of being posted.

The CR team members were provided with individual passwords to access the administration section of the program; this allowed them to view and monitor individual patients’ progress and view patient login data. If a patient had not logged in for more than 7 days, or were logging in but not progressing, they would be contacted via email or telephone by the CR specialists. All participants attended a follow-up assessment on completion of the AYH program by a CR specialist.

All data captured on the program were encrypted to safeguard patient confidentiality.

### Outcomes

#### Overview

The outcome measures employed were the Hospital Anxiety and Depression Scale (HADS), the MacNew Quality of Life (QOL) questionnaire [31], the ISWT, smoking status, diet (consumption of fruit and vegetables/day and oily fish/week), weight, Body Mass Index (BMI), and number of patients exercising for 30 minutes, 5 days per week. Baseline measurements were taken 4-6 weeks post discharge from hospital. These measurements were then repeated following completion of the AYH program.

#### Hospital Anxiety and Depression Scale

The HADS is a validated measure of anxiety and depression [32]. It consists of 14 statements, 7 for anxiety and 7 for depression. Each statement is rated on a Likert scale ranging from 0-3. A total score of 8-10 is classed as mild depression or anxiety, 11-14 as moderate depression or anxiety, and ≥15 severe depression or anxiety.

#### MacNew Quality of Life Questionnaire

The MacNew comprises 27 questions, which are divided into 3 domains: emotional, physical, and social. Each question is based on a score between 1 and 7, with lower scores corresponding to impaired QOL. This questionnaire has been reported to be both a valid and reliable measure for this population [33].

#### Incremental Shuttle Walking Test

The ISWT is a standardized field exercise test, which has shown to be both a valid and reliable test for assessing exercise capacity and has been previously reported in trials of CR [34,35]. Participants are required to walk up and down a 10-meter course at a pace increasing at 1-minute intervals. The test consists of 12 levels, with 1020 meters as the maximum that can be achieved. All participants were monitored using cardiac portable telemetry during the test. Healthy reference values have been recently described [36].

### Statistical Analysis

All data were analyzed using PASW Statistics for Windows, Version 18.0. For normally distributed data, parametric tests were carried out. Within-group changes after the AYH program in HADS scores, ISWT distance and MacNew scores, BMI and weight were analyzed using a paired *t* test. Changes in proportions after using the AYH program (eg, percentage of patients exercising for at least 30 minutes, 5 times per week; percentage of patients eating at least 2 portions of oily fish per week; percentage of patients eating at least 5 portions fruit and vegetables per day; and smoking status) were assessed using the non-parametric, paired samples McNemar’s test, as the data were categorical/binary. A *P* value of *P*≤.05 was reported as significant. Effect size was calculated as a mean change (pre-post) divided by the standard deviation of the change. The magnitude of change can be assessed against the following criteria: small, 0.2 to 0.5; moderate, 0.5 to 0.8; and large, >0.8 [37]. No formal power calculations were used as this was an observational study to assess uptake and collect data on clinical effectiveness.

### Ethics

The study was approved by Leicestershire, Northamptonshire and Rutland Research Ethics Committee 2 (approval No. 07/Q2501/114) (ID No. UHL 10322). All subjects for the study provided written informed consent.

## Results

We recruited 41 patients to the AYH program (Table 1). Patients presented with low levels of anxiety and depression and higher exercise capacity than is routinely observed in our conventional CR. Of the 41 patients who started the AYH program, 33 attended the follow-up assessment (Tables 2 and 3). There were statistically significant improvements in the ISWT (mean change 49.7 meters, SD 68.8m, *P*<.001), despite relatively high levels of performance at baseline. Statistically significant improvements were also reported for the MacNew QOL questionnaire (mean change 0.28, SD 0.4, *P*<.001). We also observed statistically significant improvements in the proportion of participants consuming 5 portions of fruit and vegetables each day: 22 (71%) to 29 (94%), *P*=.01. The proportion of those consuming at least 2 portions of oily fish each week rose from 14 (45%) to 21(68%), *P*=.01. Anxiety and depression did not change significantly. There were no significant changes to the number of patients exercising for at least 30 minutes, 5 days per week: 27 (82%) pre and 26 (79%) post. No significant changes to smoking habits or BMI were observed in the study period.

Feedback from this cohort, indicated that 22 (54%) of the 41 recruited would not have attended a traditional out-patient CR program. Participants logged on to the program between 5 and 42 times, with an average of 10 times per participant.

**Table 1:** Baseline characteristics for the participants in the pilot study (n=41).

Characteristics		Value
Age in years, mean (SD)		60.5 (11.1)
Gender, m:f		37:4
**Ethnicity, n (%)**		
	White/British	34 (82.9)
	Indian	7 (17.1)
**Initiating event, n (%)**		
	PCI	22 (53.7)
	CABG	10 (24.4)
	Other	2 (4.9)
	Valve Surgery	4 (14.6)
	Medical management	3 (7.3)
Weight in kg, mean (SD)		85.9 (12.1)
BMI in kg/m^2^, mean (SD)		28.71 (5.9)

**Table 2:** Changes in exercise performance and health status (n=33).

	Pre, mean (SD)	Post, mean (SD)	Mean change (95% CI)	*P* value	Effect size
HADS anxiety	3.7 (2.9)	4.1 (3.2)	0.4 (-0.5 to 1.2)	.41	.1
HADS depression	2.5 (2.5)	2.6 (2.6)	0.1 (-0.5 to -0.7)	.84	.0
ISWT distance	580.6 (182.9)	630.3 (178.4)	49.7 (25.3-74.1)	<.001	.7
MacNew total	5.8 (0.8)	6.1 (0.7)	0.3 (0.1-0.4)	<.001	.7
Weight, kg	85.9 (12.8)	86.2 (12.1)	0.2 (-1.3 to 1.8)	.77	.1
BMI, kg/m^2^	28.9 (6.3)	27.8 (3.2)	-1.2 (-3.3 to 1.0)	.29	.2

**Table 3:** Changes in health behaviors (n=33).

Behaviors	Pre	Post	*P* value
Exercise behavior, n (%)	27 (82)	26 (79)	1.0
Fruit and vegetables, n (%)	22 (71)	29 (94)	.01
Oily fish, n (%)	14 (45)	21 (68)	.01
Smoking, n (%)	1 (3)	0 (0)	1.0

## Discussion

### Principal Findings

This observational study demonstrates the potential benefits of using AYH as a form of CR to improve exercise capacity, quality of life, and dietary behavior in a low-risk population presenting for CR. The baseline exercise performance of this group was high, as expected in this cohort and their scope to increase their performance would be limited compared to those with lower baseline levels. The level of anxiety and depression were low at baseline, reflecting our selection criteria, again limiting the scope of improvement.

AYH may give patients a real choice of alternatives to supervised CR. Studies have shown that most patients with CHD who were still working preferred to follow a home-based rehab program to the conventional supervised CR program [5]. Alternative formats of CR, including home-based CR, such as the Heart Manual and the Angina Plan [38], have been shown to be an effective alternative to conventional CR [4]. However, these options are not widely available (5% and 2% of patients respectively in the United Kingdom [39]).

Patients require a CR program that is flexible to suit their lifestyle [40]. Using the Internet is a novel approach, providing flexibility for patients. It would potentially attract individuals who would otherwise be unable or unwilling to attend conventional CR, as traveling and the need to take time off work to attend CR classes is removed [25]. Important feedback from our patients indicated that over half (22/41, 54%) of the cohort would not have attended any other CR program. This demonstrates that there is a legitimate role for alternative formats of CR, such as AYH. Online CR has also shown to be a safe way to deliver CR [28].

Adherence to Web-based programs can be a potential issue as highlighted in previous studies [23]. Reasons for the non-compliance can vary from lack of time, to refusing to complete the program. However, several features have been identified that could help to improve adherence to a Web-based program. Making the program tailored to the user and interactive [41] as well as allowing users to set their own personal goals [42] have been shown to help improve adherence to Web-based interventions. The AYH program has incorporated all these features. The program is tailored to the individual, identifying their specific risk factors for CHD, providing interaction and self-monitoring. Self-monitoring is particularly beneficial as it has been shown to help increase an individual’s awareness of their condition [43], thus allowing them to take better control of their condition.

In order for any Web-based program to be successful, it must suit the working practices of the health care professional (HCP) [44]. This was acknowledged when developing the AYH program, ensuring that it can be easily incorporated into the CR service and does not increase the workload of the HCP. Each member of the CR team was provided with their own password to log onto an administration section of the program, allowing them to enroll patients on to the program, monitor their progress, and follow up with those who were not logging on or not progressing, via either email or phone call. The private messaging facility on the AYH program allowed for an easy and convenient way for the patient to access a CR specialist and vice versa. The ability to easily communicate with a large number of patients could have the added benefit of saving time and cost [45].

Following the pilot study, AYH has been incorporated into the CR service and made available to patients referred to the CR department. The criteria for inclusion were amended to allow for moderate risk patients to take part, that is, patients who completed at least level 5 (250 meters) of the ISWT and who had normal to moderate left ventricular dysfunction. We have collected data from 106 participants, using the outcomes employed in this study, which showed that after following AYH, there were statistically significant changes (mean change 47.16 m, *P*<.001) in exercise capacity, QOL (*P*<.001), and in a larger group, improvements in the number of patients exercising for at least 30 minutes, 5 days per week (increasing from 69.8% to 84.0%, *P*=.001). Statistically signifcant improvements were also noted for anxiety (*P*=.01) and depression (*P*=.03) and for those eating at least 2 portions of oily fish per week (*P*=.02). However, no changes were reported for consumption of fruit and vegetables or smoking habits. Feedback from this cohort indicated that over two-thirds of the participants (69/106, 65%) would not have attended a traditional out-patient CR program.

### Limitations

The data reported are our initial findings from the AYH program; we now need to proceed to a clinical effectiveness trial incorporating a control group. Furthermore, the program was not offered to high-risk patients. Participants recorded low anxiety and depression scores and relatively high ISWT at baseline; nevertheless, statistically significant improvements were still observed.

**Figure 4:**
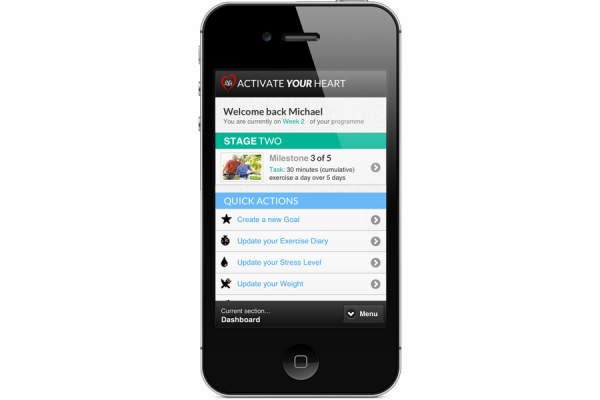
Mobile version of the Activate Your Heart dashboard.

**Figure 5:**
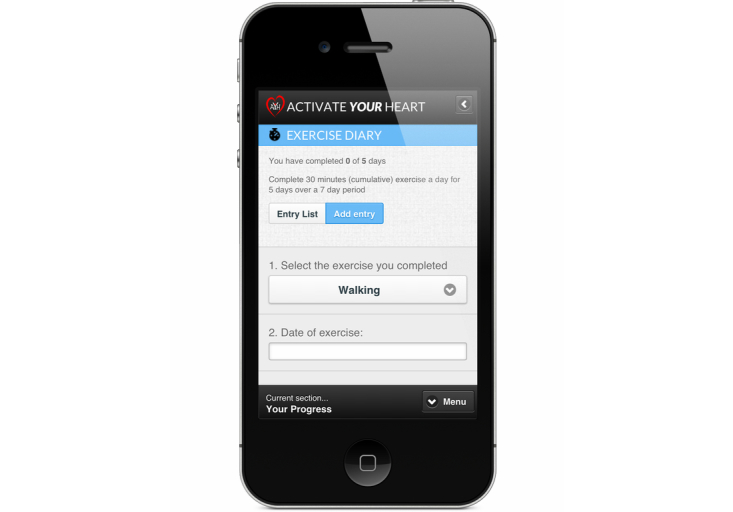
Adding exercise using a smartphone.

In order to make AYH more accessible, a mobile version of AYH has also been developed. This allows users to access the program on their mobile device, such as smartphones and tablets (Figures 4 and 5). Using mobile phones to access CR has been shown to motivate patients to achieve their goals and to increase adherence [10]. A recent study [46] illustrated how sending emails to users helped increase adherence to the program. The latest version of AYH now creates automatic emails that are sent based on a user’s level of interaction, for example, those not progressing or not logging on to the program for more than 7 days are flagged and emails are automatically sent to them.

### Conclusions

AYH has been designed to support individuals with CHD, promoting an alternative form of CR and provides a viable alternative option for patients, as well as providing a timely and patient-centered approach to CR. AYH enables patients to choose a program that best suits their lifestyle. The convenience of using AYH means that many of the barriers associated with conventional CR programs are removed. To our knowledge, AYH is the first online comprehensive CR program to be introduced into a clinical service. This paper demonstrates that AYH can positively influence exercise capacity, QOL, and dietary behavior in a low-risk group and can be integrated into an existing CR service.

## Abbreviations

AYH: Activate Your Heart
BMI: body mass index
CABG: coronary artery bypass graft
CHD: coronary heart disease
CR: cardiac rehabilitation
HADS: Hospital Anxiety Depression Score
HCP: health care professional
ISWT: Incremental Shuttle Walk Test
PCI: percutaneous coronary intervention
QOL: Quality of Life
